# Draft Genome Sequence of Bifidobacterium longum UMA026, Isolated from Holstein Dairy Cow Feces

**DOI:** 10.1128/genomeA.00559-18

**Published:** 2018-06-21

**Authors:** Korin Albert, David A. Sela

**Affiliations:** a Department of Food Science, University of Massachusetts, Amherst, Massachusetts, USA; b Molecular and Cellular Biology Graduate Program, University of Massachusetts, Amherst, Massachusetts, USA; c Department of Microbiology, University of Massachusetts, Amherst, Massachusetts, USA; d Department of Microbiology and Physiological Systems, University of Massachusetts Medical School, Worcester, Massachusetts, USA

## Abstract

The draft genome sequence of an isolate identified as Bifidobacterium longum is communicated herein. This strain was isolated from the feces of a 1-week-old Holstein dairy cow. The draft genome of this Bifidobacterium longum isolate is 2.39 Mb in length, with a G+C content of 60.1%.

## GENOME ANNOUNCEMENT

Freshly obtained bovine feces were mixed with 5 ml of sterile peptone water and introduced to Bifidobacterium-specific medium (BSM) agar to select for bifidobacteria. This medium consists of 55 g/liter de Man-Rogosa-Sharpe agar, 15 g/liter agar, 0.05% (wt/vol) l-cysteine (Sigma-Aldrich), and 0.05% (wt/vol) mupirocin (AppliChem Panreac) (1). Agar plates were incubated at 37°C for 24 h in an anaerobic chamber maintained with a gas mix of 7% H_2_, 10% CO_2_, and N_2_ for balance (Coy Laboratory Products, Grass Lake, MI). Individual colonies were selected and grown in BSM broth for 12 h under the same conditions. Liquid cultures were preserved as freezer stocks at −80°C using a 25% glycerol solution.

Genomic DNA was extracted using the MasterPure Gram-positive DNA purification kit (Epicentre [an Illumina Company], Madison, WI). The sequencing library was prepared using the Nextera XT 150-bp paired-end library preparation kit (Illumina, San Diego, CA). Subsequently, whole-genome sequencing of the isolate was performed on the Illumina NextSeq platform with v2 reagents. Reads were assembled *de novo* using SPAdes version 3.9.1, and the resulting assembly was run through Pilon to correct minor errors (2, 3). Gene model prediction and annotation were performed using the Rapid Annotation using Subsystem Technology (RAST) annotation service (4–6).

The 16S rRNA marker gene sequence of the isolate designated UMA026 exhibits greater than 99.2% similarity to all subspecies belonging to the Bifidobacterium longum taxon (7). In order to further resolve the identity of UMA026, a multilocus sequence typing approach was employed using concatenated sequences of four conserved housekeeping genes, 16S rRNA gene, *clpC*, *xfp*, and *dnaJ*. This approach placed the phylogenetic position of UMA026 within the Bifidobacterium longum subsp. *suis*/*suillum* clade (8). Despite not being fully characterized, this clade incorporates B. longum strains that are isolated from nonhuman mammals. Thus, UMA026 is clearly a B. longum strain, with a phylogeny consistent with its dairy cow host.

### Accession number(s).

This whole-genome shotgun project has been deposited at DDBJ/ENA/GenBank under the accession number PHUM00000000. The version described in this paper is version PHUM01000000.
